# Non-rigid Multi-Modal Medical Image Registration Based on Improved Maximum Mutual Information PV Image Interpolation Method

**DOI:** 10.3389/fpubh.2022.863307

**Published:** 2022-06-01

**Authors:** Liting He

**Affiliations:** School of Computer and Information Science, Southwest University, Chongqing, China

**Keywords:** PV image interpolation method, Shannon entropy, non-rigid medical image registration, DFP algorithm, CT-MRI-PET image

## Abstract

With the continuous improvement of medical imaging equipment, CT, MRI and PET images can obtain accurate anatomical information of the same patient site. However, due to the fuzziness of medical image physiological evaluation and the unhealthy understanding of objects, the registration effect of many methods is not ideal. Therefore, based on the medical image registration model of Partial Volume (PV) image interpolation method and rigid medical image registration method, this paper established the non-rigid registration model of maximum mutual information Novel Partial Volume (NPV) image interpolation method. The proposed NPV interpolation method uses the Davidon-Fletcher-Powell algorithm (DFP) algorithm optimization method to solve the transformation parameter matrix and realize the accurate transformation of the floating image. In addition, the cubic B-spline is used as the kernel function to improve the image interpolation, which effectively improves the accuracy of the registration image. Finally, the proposed NPV method is compared with the PV interpolation method through the human brain CT-MRI-PET image to obtain a clear CT-MRI-PET image. The results show that the proposed NPV method has higher accuracy, better robustness, and easier realization. The model should also have guiding significance in face recognition and fingerprint recognition.

## Introduction

Using artificial intelligence technology to collate and analyze medical images (1–4) can improve medical and health services (5) and alleviate the imbalance of medical and health resource (6–9) allocation, which is the focus of the development of medical artificial intelligence. Many previous studies have mentioned that the application of artificial intelligence in the field of smart medicine should be deepened, new models and means of artificial intelligence diagnosis and treatment (10) should be promoted, and a fast and accurate intelligent medical system (11) should be established. As the main component of smart medicine, medical image plays an increasingly important role in assisting medical diagnosis and promoting the rationalization of medical decision-making. Common medical images include X-ray, computed tomography (CT) (12), magnetic resonance imaging (MRI) (13, 14), positron emission tomography (PET) (15) and single-photon emission computed tomography (SPECT) (15). Medical image registration (16–19) is a basic task in the process of medical image processing. In clinical practice, it is usually necessary to perform multiple imaging in multiple modes or in the same mode for the same patient, that is, to obtain information from several images and conduct comprehensive analysis. The image registration technology (20–23) is used to fuse the above images, and the multi-faceted information of the human body is expressed on the same image at the same time. The internal structure and functional state of the human body are reflected from the medical image (24–27), and the anatomical and physiological pathological information of the human body is more directly provided, thus playing the role of multi-information visualization at the same time.

After years of development, computer in-depth learning technology (28) and artificial intelligence technology (29) have made significant breakthroughs in theory and practice. They have made revolutionary progress in realizing the open sharing of medical information and using artificial intelligence to organize and analyze fragmented medical information. The combination of image fusion analysis and radiology is of great significance for understanding complex diseases and realizing accurate judgment, and is of great help for proposing the best diagnosis results and treatment schemes. In the process of imaging diagnosis (30, 31), single image data often cannot fully display the pathological structure of patients, and pathological features need to be expressed by multi-directional image features. Compared with a single sensor, multi-sensor (32, 33) can provide more extensive data for better identification of tissue or lesion details.

Some scholars focus on the improvement of fusion methods, such as Xiao and Pedrycz (34) and Xiao (35) proposed fuzzy value decision theory and extended it to the complex domain. Other scholars focus on improving interpolation algorithms, such as in 2005, Xiao (36) proposed a qualitative perturbation PV interpolation algorithm to avoid local extremum on grid points and non-grid points. In 2010, Xiao (37) proposed an improved Blackman-Harris PV (BHPV) interpolation algorithm, and introduced the Blackman-Harris window sinc function to replace the kernel function of the traditional PV interpolation. He can improve the extreme value problem of the traditional method. In 2021, Zang et al. (38) proposed an improved PV interpolation method to calculate the chord function. However, these methods do not pay much attention to the characteristics of the model itself. Therefore, how to fuse multiple image information with minimum complexity cost to obtain high reliability registration results is the primary problem to be solved in medical image registration.

To solve this problem, through the analysis and research on rigid medical image registration based on the maximum mutual information PV (Partial Volume) (39) image interpolation method, this paper proposes a decision fusion model for multiple information image data, namely the non-rigid registration model based on the maximum mutual information NPV (Novel Partial Volume) image interpolation. The cubic B-spline interpolation method can obtain better decision results under various uncertain information conditions. In addition, the effect is particularly significant in image-assisted diagnosis and treatment.

The main contributions of this paper are listed below:

- The proposed NPV method combines cubic spline with DFP algorithm for the first time, and introduces image registration to make the registration result more reasonable and reliable.
- By comparing the synthetic image with three original images to be registered, the image registration method proposed in this paper has great advantages in comprehensively displaying pathological details, balancing the advantages of each image, and matching the corresponding spatial points.
- The improved method proposed in this paper is the traditional maximum mutual information PV image interpolation method based on gray information, so that the registration model does not need to segment the image. It is simple, accurate and robust.

The organizational structure of this paper is as follows. The second chapter focuses on the problems to be solved and the relevant implementation steps of the NPV interpolation method. In the third chapter, some examples are introduced to prove the effectiveness of the proposed method. In the fourth chapter, the full text is summarized.

## Preliminaries

### PV Image Interpolation

**Figure 1:**
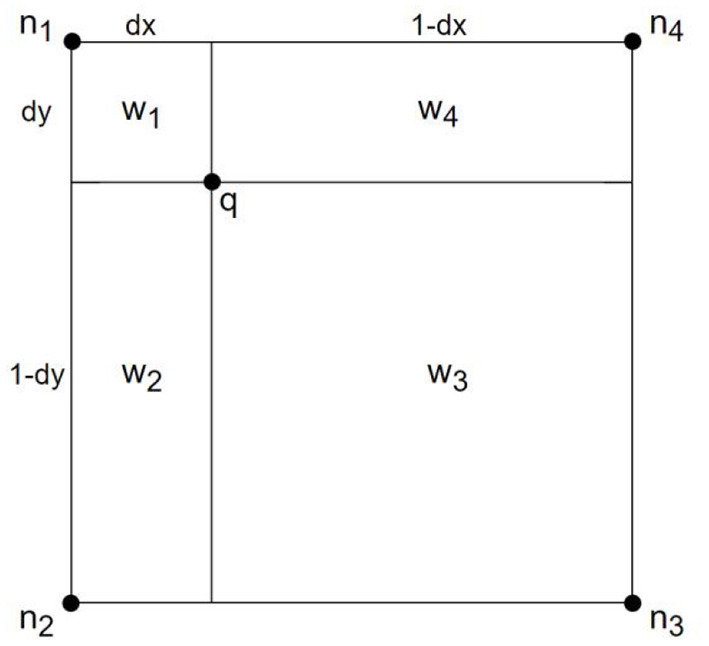
PV interpolation image.

PV image interpolation (40) is a common registration method in medical research, which is convenient in updating the joint histogram of two images. Suppose that the reference image is R, and the image to be registered is F for any point *p* on image F. After transforming *T*, the point *q* in the reference image R is corresponding, that is, *q* = *T*(*p*). The adjacent pixels of *q* are *n*_1_, *n*_2_, *n*_3_, and *n*_4_, respectively, as shown in Figure 1.

***Definition 1:*** Let the joint histogram be *h*, the remaining parameters are shown in Figure 1.

(1) $$\begin{array}{r} \underset{i}{\sum }W_{i}=1,i=1,2,3,4, \end{array}$$

(2) $$\begin{array}{r} \forall_{i}h_{rm}\left[r\left(x\right),f\left(n_{i}\right)\right]+=w_{i}, \end{array}$$

(3) $$\begin{array}{r} w_{1}=\left(1-dx\right)\times \left(1-dy\right), \end{array}$$

(4) $$\begin{array}{r} w_{2}=dx\times \left(1-dy\right), \end{array}$$

(5) $$\begin{array}{r} w_{3}=dx\times dy, \end{array}$$

(6) $$\begin{array}{r} w_{4}=\left(1-dx\right)\times dy. \end{array}$$

**Figure 2:**
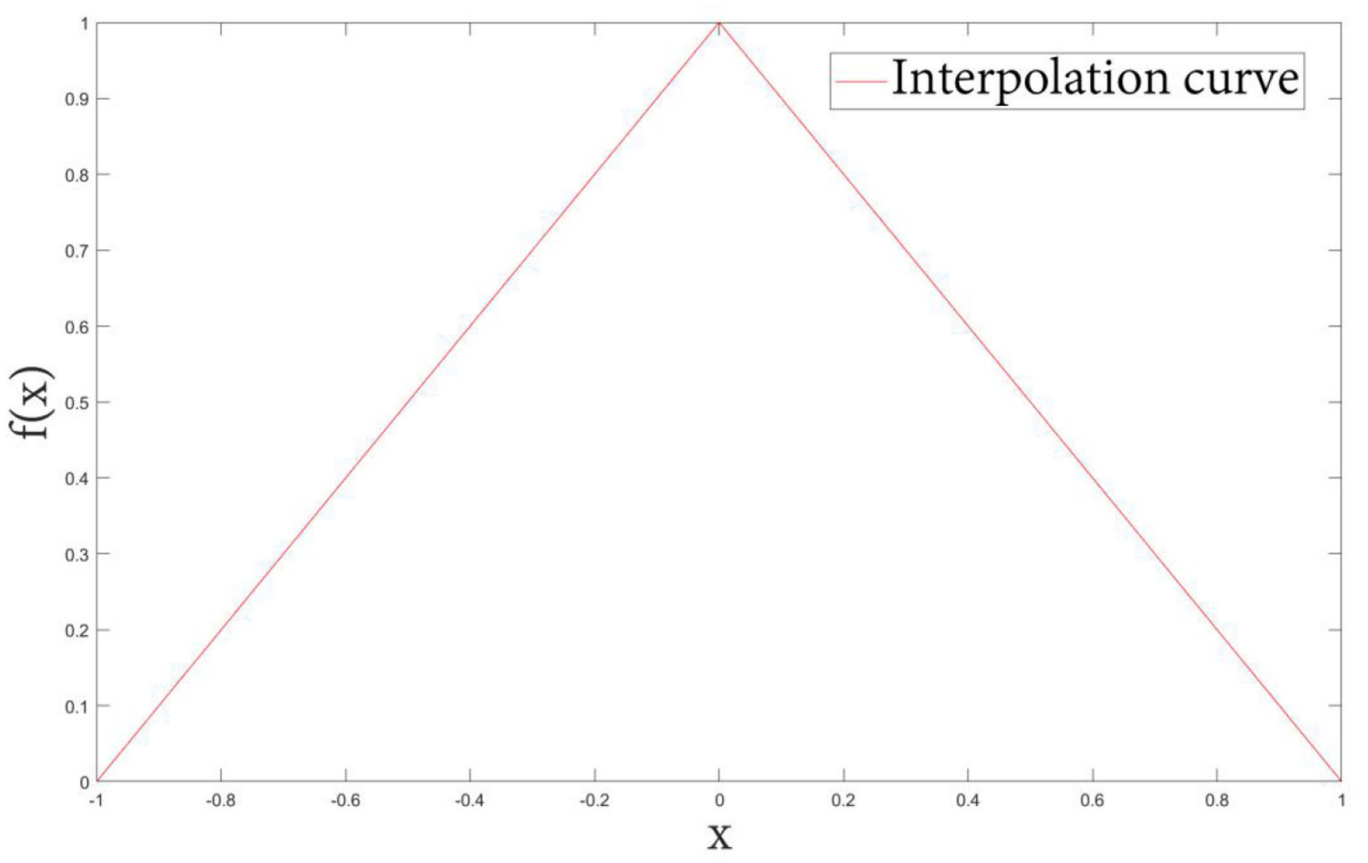
Basic kernel curve.

***Definition 2:*** Let *f*(*x*) be the classical PV interpolation kernel function plotted in Figure 2, defined as:

(7) $$\begin{array}{r} f\left(x\right)=\{\begin{array}{c} 1-|x|,|x|\;<1\\ 0,|x|\;\geq 1 \end{array}. \end{array}$$

In PV interpolation, the value of each gray pair in the joint histogram is the sum of several decimals with small changes, so it not only makes the calculated mutual information value more accurate, but also smooths the change of the mutual information curve when the registration has a small transformation.

**Figure 3:**
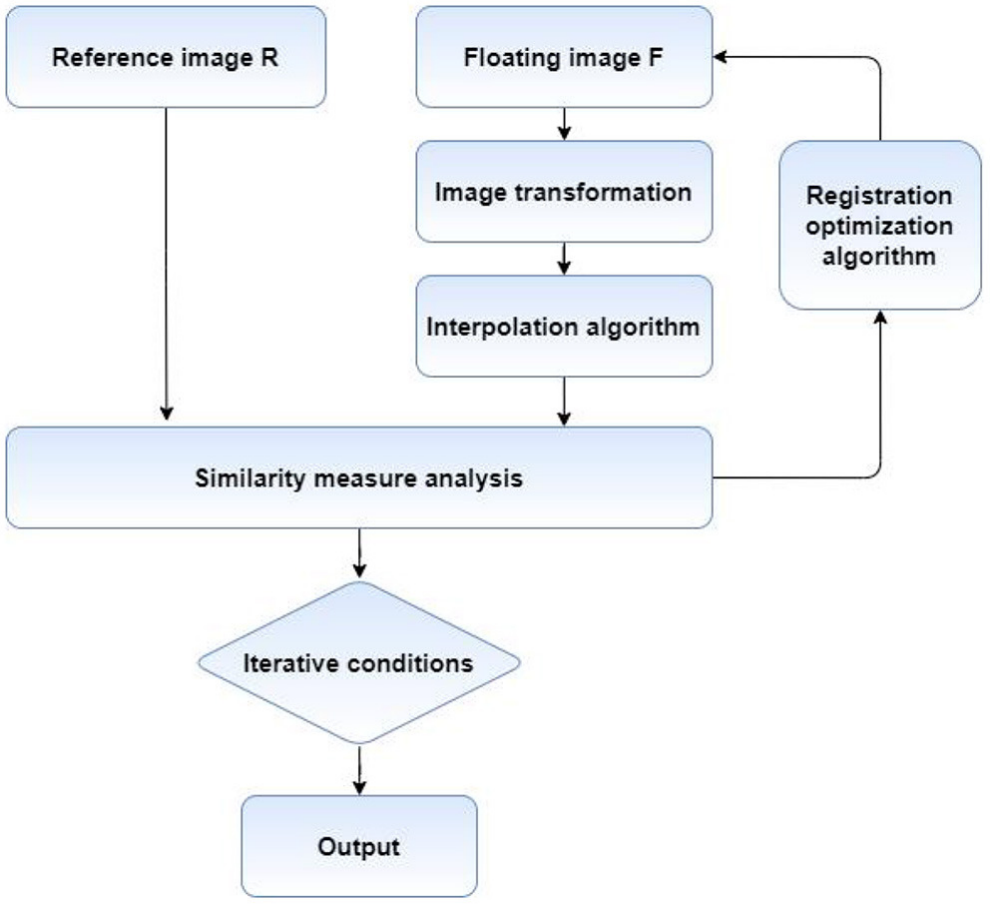
The flowchart of PV image interpolation method.

The process of medical image registration mainly includes four key parts, graphic transformation, registration optimization method, deformation image interpolation method and similarity measure analysis. The flow chart is shown in Figure 3. First of all, it is necessary to find the appropriate graphic transformation model and select the appropriate optimization method to solve the equation. Then, the floating image is transformed on the results obtained by solving, and the deformed image is interpolated. Finally, the similarity measure of the two images is calculated, and whether the next iteration is needed according to the similarity degree.

## Proposed Methods

The registration process based on image intensity is actually an optimization problem, that is, after selecting the appropriate similarity measure for the image to be registered, the similarity measure between images can be maximized as far as possible. The registration process is mainly composed of five parts, image preprocessing, image transformation, gray interpolation, objective function optimization, and similarity measure calculation. When achieving high-precision alignment of medical images, we select the reference image CT as the reference image, and select PET and MRI as floating image information sources to provide information.

**Figure 4:**
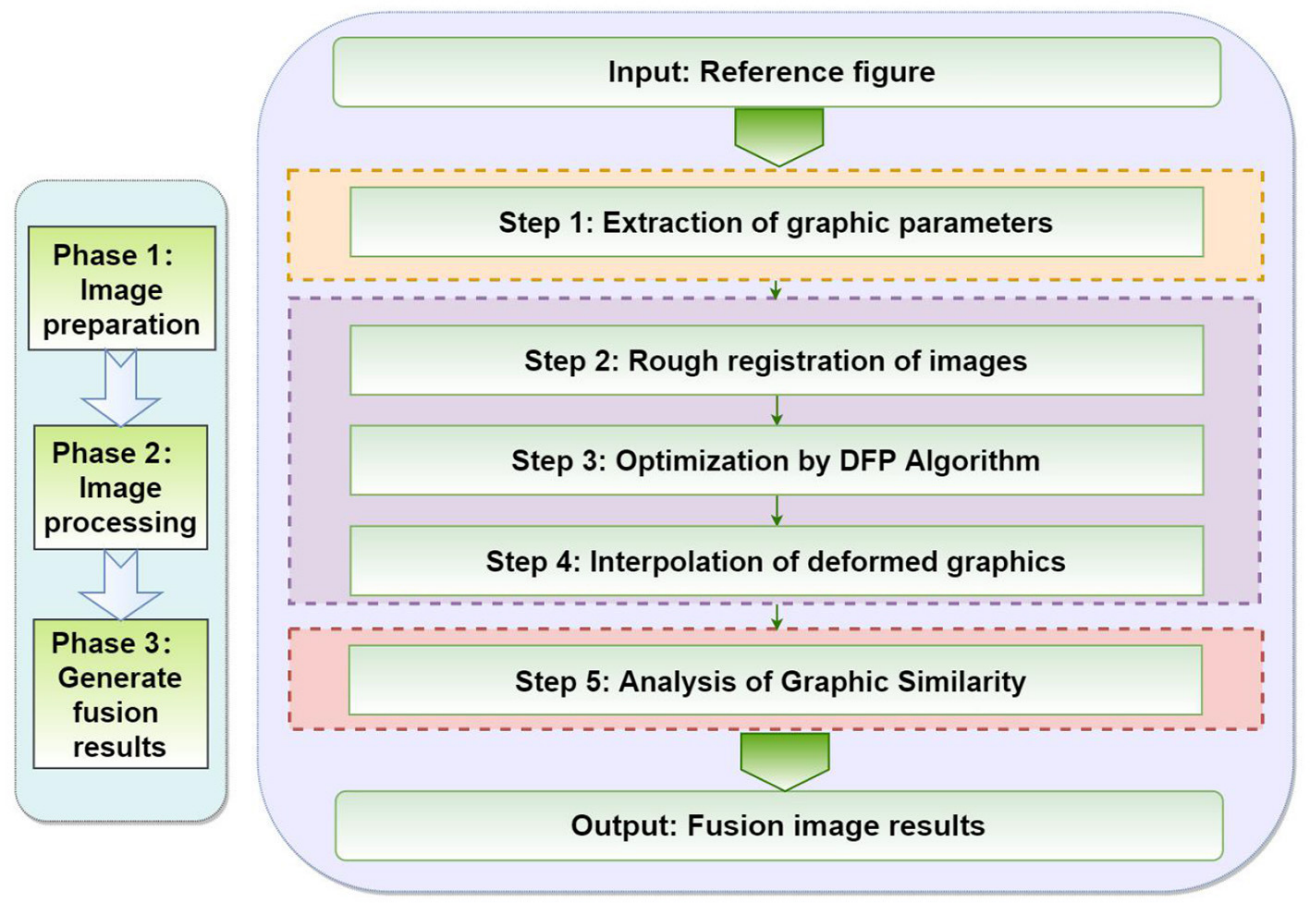
Procedure followed by the proposed method.

In this chapter, we will focus on the five steps of the proposed method, as shown in Figure 4. First of all, we need to extract the parameter values of the graphics. The second step of image transformation is necessary in the coarse registration. In the third step, the DFP optimization algorithm is used to optimize the image. In the fourth step, the deformed graphics are interpolated. Finally, the similarity analysis of the graphics is completed and the results are output.

### Preliminaries

Coordinate origin is defined in the gray center of the image, the coordinates are (*c*_*x*_, *c*_*y*_, *c*_*z*_), *g*(*x, y, z*) is the gray value of point (*c*_*x*_, *c*_*y*_, *c*_*z*_).

(8) $$\begin{array}{r} c_{x}=\frac{\underset{x}{\sum }xg\left(x,y,z\right)}{\underset{x}{\sum }g\left(x,y,z\right)},c_{y}=\frac{\underset{y}{\sum }yg\left(x,y,z\right)}{\underset{y}{\sum }g\left(x,y,z\right)},c_{z}=\frac{\underset{z}{\sum }zg\left(x,y,z\right)}{\underset{z}{\sum }g\left(x,y,z\right)}. \end{array}$$

For the images to be registered, in order to improve the registration accuracy, it is necessary to remove the interference such as mask and bed frame in the image. In order to improve the registration speed and balance the relationship between the registration speed and accuracy, some voxel points are usually selected as the sample set of the objective function, namely, the images to be registered are sampled. In this paper, regular grid spacing is used for sampling. Several individual pixels are selected from CT, MRI, and PET images to be registered as sample sets.

### Image Transformation

The key to register multiple different modal images is to find a suitable transformation model so that all pixels on the floating image can take you through the transformation model to find the unique pixel coordinates corresponding to them in the reference image and maintain the same or similar anatomical information. In this paper, rigid transformation is used for global coarse registration and then non-rigid transformation is used for local elastic registration.

(1) Rigid transformation based on affine transformation

Rigid transformation mainly includes linear transformation and affine transformation. The affine transformation has the ability to map the floating image line to the line in the reference image space and ensure the parallel relationship between different lines, and it can be expressed as a combination of linear transformations such as rotation, scaling and translation. Therefore, this paper uses affine transformation method for coarse registration of CT, MRI, and PET images.

Assuming that the pixel point on the original image is the spatial coordinate $p_{0}(x_{0}{}^{\prime },y_{0}{}^{\prime },z_{0}{}^{\prime })$ after *p*_0_(*x*_0_, *y*_0_, *z*_0_) translation transformation, the affine transformation can be expressed as:

(9) $$\begin{array}{r} \left[\begin{array}{c} {x_{0}}^{\prime }\\ {y_{0}}^{\prime }\\ {z_{0}}^{\prime }\\ 1 \end{array}\right]=\left[\begin{array}{cccc} a_{11} & a_{12} & a_{13} & 0\\ a_{21} & a_{22} & a_{23} & 0\\ a_{31} & a_{32} & a_{33} & 0\\ 0 & 0 & 0 & 1 \end{array}\right]\left[\begin{array}{c} x_{0}\\ y_{0}\\ z_{0}\\ 1 \end{array}\right]. \end{array}$$

(2) Non-rigid transformation based on cubic B-spline

For a three-dimensional floating image Ω = {(*x, y, z*)|0 ≤ *x* ≤ *X*, 0 ≤ *y* ≤ *Y*, 0 ≤ *z* ≤ *Z*}, the control points ϕ_*i,j,k*_ in the image constitute the size of the grid *n*_*x*_, *n*_*y*_, *n*_*z*_, and limit its movement within the range of four grid points in its neighborhood, as below:

(10) $$\begin{array}{r} n_{x}=\frac{X}{\lambda_{x}},n_{x}=\frac{Y}{\lambda_{y}},n_{x}=\frac{Z}{\lambda_{z}}. \end{array}$$

Among them, λ_*x*_, λ_*y*_, λ_*z*_ represents the distance of grid points on the *x, y, z* axis, and selects different sizes according to different registration requirements. Usually, the number that can divide the image size *X, Y, Z* is selected to reduce the computational complexity and ensure the stability of image transformation. The single transformation *T* is used to transform the spatial coordinates of the control point to (Δ*x*, Δ*y*, Δ*z*), and the coordinate transformation of all points in the neighborhood of the control point (*x, y, z*) is fitted with the change of the control point, which is expressed as (*x* + Δ*x, y* + Δ*y, z* + Δ*z*). The mathematical expression of the transformation is as follows.

(11) $$\begin{array}{r} T\left(x,y,z\right)=\overset{3}{\underset{i=0}{\sum }}\overset{3}{\underset{m=0}{\sum }}\overset{3}{\underset{n=0}{\sum }}B_{i}\left(u\right)B_{m}\left(w\right)\phi_{i+l,j+m,k+n}. \end{array}$$

Among them, *i, j, k* is the index of the control point coordinates (Δ*x*, Δ*y*, Δ*z*) in the grid, *u, v, w* is the distance between the corresponding point and the integer coordinate point, *l, m, n* is the number of B-spline basis functions, *B*_*l*_, *B*_*m*_, *B*_*n*_ represents *l, m, n* basis functions, respectively. The calculation formulas of each parameter are as follows:

(12) $$\begin{array}{r} \{\begin{array}{c} i=\frac{x}{\lambda_{x}}-1\\ j=\frac{y}{\lambda_{y}}-1\\ k=\frac{z}{\lambda_{z}}-1 \end{array} \end{array}$$

(13) $$\begin{array}{r} \{\begin{array}{c} u=x-\left\lfloor x\right\rfloor \\ v=y-\left\lfloor y\right\rfloor \\ w=z-\left\lfloor z\right\rfloor \end{array}. \end{array}$$

where ⌊*x*⌋, ⌊*y*⌋, ⌊*z*⌋ represents an integer not greater than *x, y, z*. The expressions of zeroth to cubic B-spline basis functions are as follows.

(14) $$\begin{array}{r} \{\begin{array}{c} B_{0}\left(u\right)=\frac{{\left(1-u\right)}^{3}}{6}\\ B_{1}\left(u\right)=\frac{3u^{3}-6u^{2}+4}{6}\\ B_{2}\left(u\right)=\frac{-3u^{3}+3u^{2}+3u+1}{6}\\ B_{3}\left(u\right)=\frac{u^{3}}{6} \end{array} \end{array}$$

### DFP Optimization Algorithm

The transformation matrix is essentially an optimization problem for solving multi-parameter objective function. The use of DFP in the registration process can make the calibrated image more optimized.

In the registration process, the cubic B-spline transformation function is used as the objective function, and the transformation parameters are solved by DFP algorithm. The specific steps are as follows:

1. Given parameters δ ∈ (0, 1), σ ∈ (0, 0.5), initial point $x_{0}\epsilon {\mathbb{R}}^{n}$, termination error 0 ≤ ε ≤ 1. Initially symmetric positive definite matrix *H*_0_ and let *k* = 0.
2. Calculate *g*_*k*_ = ∇*f*(*x*_*k*_), when ||*g*_*k*_|| ≤ ε stops, output *x*_*k*_ as the approximate minimum point.
3. Calculate the search direction *d*_*k*_ = −*H*_*k*_*g*_*k*_.
4. Let *m*_*k*_ be the minimum non-negative integer m satisfying the following inequality.

(15) $$\begin{array}{r} f\left(x_{k}+\delta^{m}d_{k}\right)\leq f\left(x_{k}\right)+\sigma \delta^{m}g_{k}^{T}d_{k}. \end{array}$$

Let $\alpha_{k}=\delta^{mk},x_{k+1}=x_{k}+\alpha_{k}d_{k}$, then calculate *s*_*k*_, *y*_*k*_;

(5) From Equation (16) calculate *H*_*k*+1_;

(16) $$\begin{array}{r} H_{k+1}=\{\begin{array}{c} H_{k},\;{s_{k}}^{T}y_{k}\leq 0\\ H_{k}-\frac{H_{k}y_{k}{y_{k}}^{T}H_{k}}{{y_{k}}^{T}H_{k}y_{k}}+\frac{s_{k}{s_{k}}^{T}}{{s_{k}}^{T}y_{k}},{y_{k}}^{T}s_{k}>0 \end{array}. \end{array}$$

(6) Let *k* = *k* + 1, turn to Step 1.

### Image Interpolation Algorithm After Deformation

Since the reference image and floating image are discrete image data, the pixel coordinates are integers, and the position of the pixel coordinates of the image obtained by graphic transformation is not necessarily exactly located in the integer coordinates. Therefore, it is inevitable to interpolate the pixels on the transformed image. Interpolation belongs to a resampling method of the image. It not only needs to consider the image accuracy after interpolation, but also needs to consider the computational complexity and the protection of the original image data. PV and trilinear interpolation methods are the most widely used in interpolation calculation based on mutual information registration. In the three-dimensional case, the objective function *T* = *f*(*x*) satisfies the following conditions for the point *m*(*x*_*i*_, *x*_*j*_, *x*_*k*_) of floating image MRI and the point *n*(Y_*i*_ + η_*j*_, Y_*j*_ + η_*j*_, Y_*k*_ + η_*k*_) of reference image CT.

(1) *T*(*x*) ≥ 0, *x* ∈ *Z*;

(2) $\overset{+\infty }{\underset{m=-\infty }{\sum }}f\left(m-\eta \right)=1,m\in Z,0\leq \eta <1$

**Figure 5:**
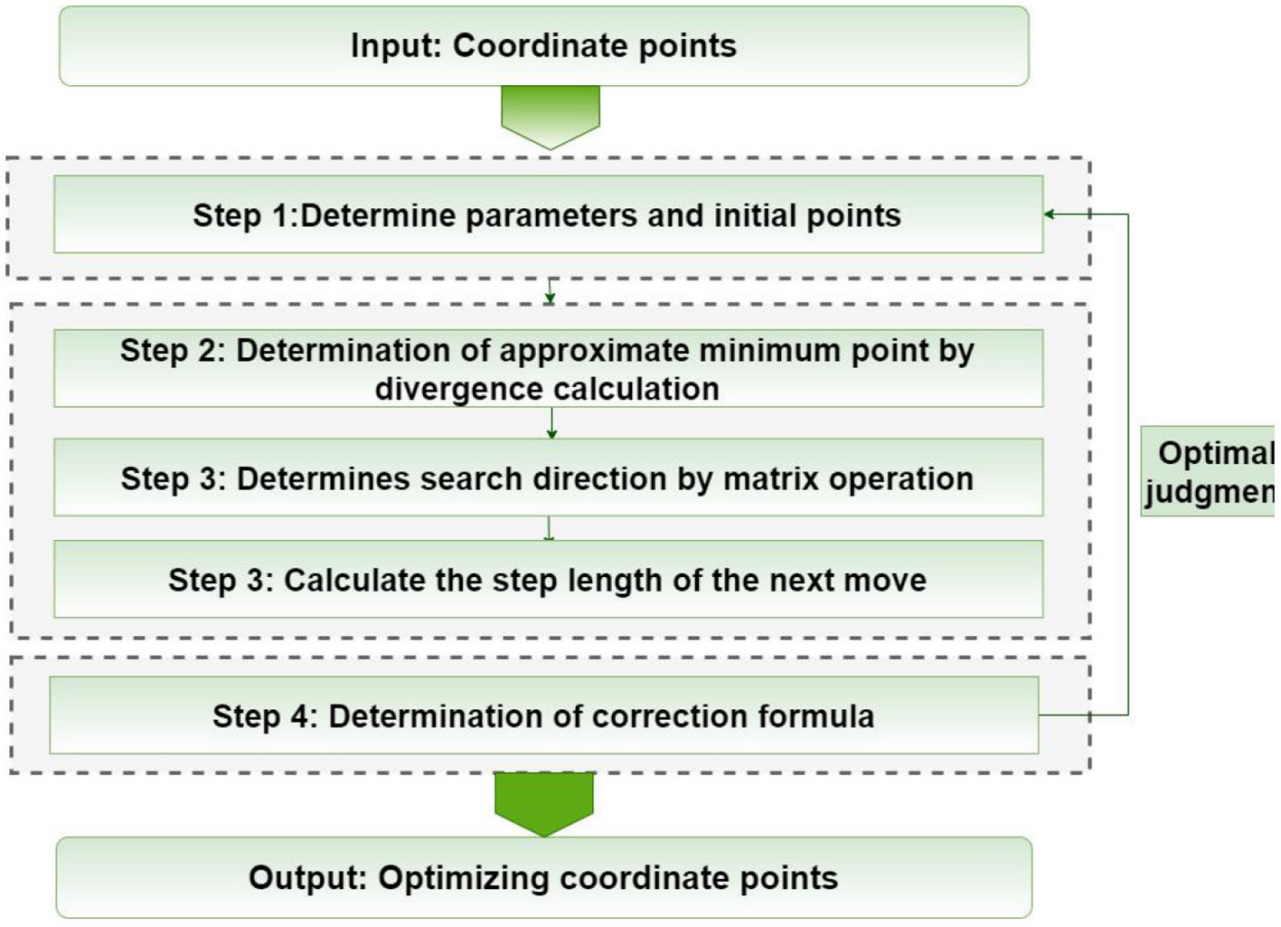
Flow chart of DFP optimization algorithm.

Because cubic spline satisfies the above two conditions at the same time, using cubic spline as kernel function *f*(*x*) as below, plotted in Figure 5.

(17) $$\begin{array}{r} f\left(x\right)=\{\begin{array}{c} ax^{3}+bx^{2}+cx+d,|x|<1\\ 0,otherwise \end{array} \end{array}$$

And the gray contribution histogram can be obtained as follows:

(18) $$\begin{array}{r} h\left[F\left(x_{i},x_{j},x_{k}\right),R\left(y_{i}+p,y_{j}+q,y_{k}+r\right)\right]+=f_{1}\left(p-\eta_{i}\right)\\ f_{2}\left(q-\eta_{j}\right)f_{3}\left(r-\eta_{k}\right). \end{array}$$

where *p, q, r* ∈ (0, 1) as an integer.

With the increase of kernel function order, the local extremum generated by interpolation is eliminated. However, the computational complexity will be more when the order is >3. Therefore, considering the registration time benefit and accuracy, cubic spline is selected for gray histogram calculation.

**Figure 6:**
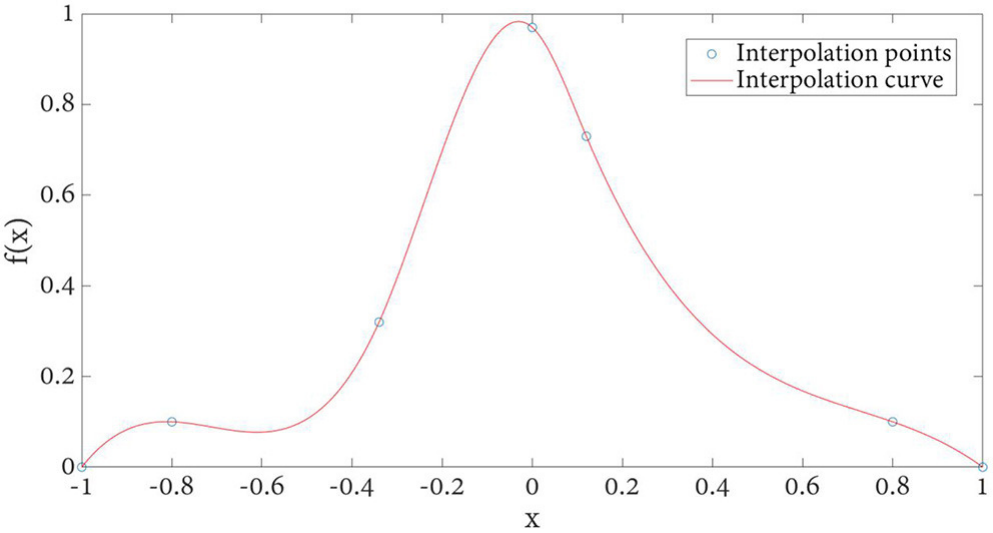
Cubic B-spline interpolation kernel function interpolation curve.

The kernel function obtained by cubic spline interpolation method is shown in Figure 6. Compared with the basic image interpolation method (Figure 2), the image interpolation curve obtained by the improved method is smoother, which effectively avoids the occurrence of burrs and sharp points. Especially, the proposed NPV method has better accuracy for changing smaller points.

### Similarity Measure Analysis

Similarity measure is the consideration of calculating the similarity between reference image and floating image. This paper uses the similarity measure based on gray level, which includes the sum of squares of error, mean square error, mutual information and other methods. Since mutual information is an automatic calculation method, in order to improve the registration speed, this paper uses mutual information method to analyze the similarity measure.

The calculation of mutual information is based on Shannon entropy, which is defined as follows.

(19) $$\begin{array}{r} H=\underset{i}{\sum }p_{i}\log\frac{1}{p_{i}}=-\underset{i}{\sum }p_{i}\log p_{i}. \end{array}$$

where *p*_*i*_ represents the probability of information appearing. The mutual information between reference image CT and floating image MRI is as follows.

(20) $$\begin{array}{r} H\left(R_{CT}\right)=-\underset{r}{\sum }p_{R}\left(r\right)\log p_{R}\left(r\right). \end{array}$$

(21) $$\begin{array}{r} H\left(F_{MRI}\right)=-\underset{f}{\sum }p_{F}\left(f\right)\log p_{F}\left(f\right). \end{array}$$

The joint entropy of two images is defined as follows.

(22) $$\begin{array}{r} H\left(R_{CT},F_{MRI}\right)=-\underset{r,f}{\sum }p_{PF}\left(r,f\right)\log p_{RF}\left(r,f\right). \end{array}$$

$NMI\left(R,F\right)=\frac{H\left(R_{CT}\right)\;+\;H\left(F_{MRI}\right)}{H\left(R_{CT},F_{MRI}\right)}$ is used to calculate mutual information, and the registration is completed when mutual information reaches the maximum.

## Examples

**Figure 7:**
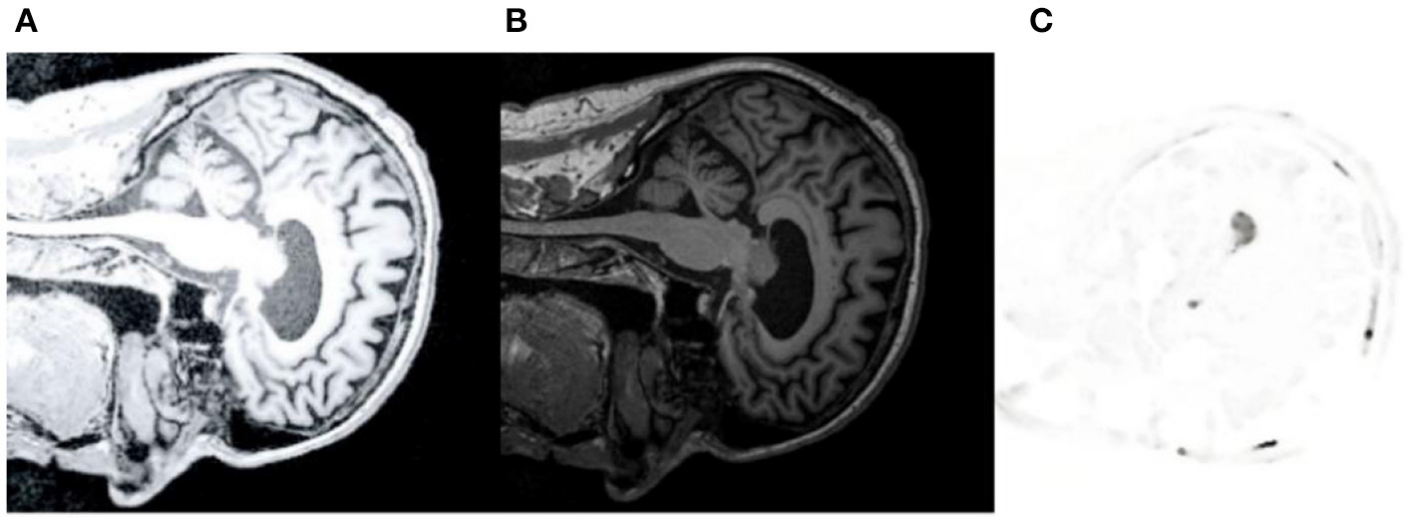
CT/MRI/PET reference image. **(A)** shows CT reference data images. **(B)** is MRI reference data image. **(C)** is PET reference data image.

The brain CT, MRI, and PET images with AD were extracted from ANDI and OASIS databases for analysis. And we used Python to analyze and extract the data. Figure 7A is the CT reference image, Figure 7B is MRI reference data, and Figure 7C is PET reference data images. Medical image preprocessing includes image gray normalization, removing interference background and extracting region of interest. Define a three-dimensional coordinate system for two images to be registered. Taking CT as reference image R and MRI as floating image F, the gray level of body pixels on two images from different devices is normalized to 256 gray level.

**Figure 8:**
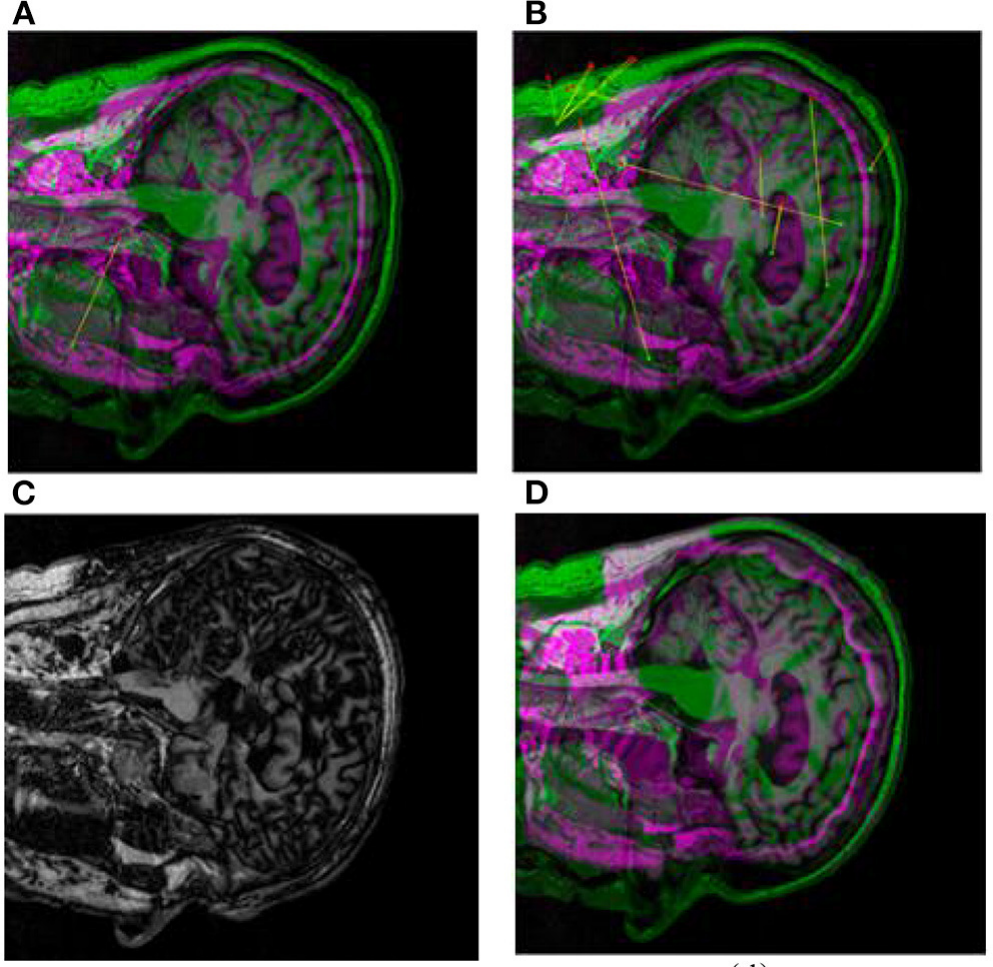
Registration images of brain CT and MRI. **(A)** shows the pre-processed initial registration image. **(B)** medical images after multi-modal registration. **(C)** is affine transformation global rough registration of medical images. **(D)** represents medical image with high precision registration after non-rigid transformation.

First, we use CT images as reference images and MRI images as floating images for medical registration. In Figures 8A–D are the segmented registration images of CT and MRI images, respectively. In Figure 8A, the initial registration image is preprocessed. Figure 8B is the medical image after multimodal registration. Figure 8C is a medical image of affine transformation global coarse registration. Figure 8D shows the medical image after high-precision registration of non-rigid transformation.

**Figure 9:**
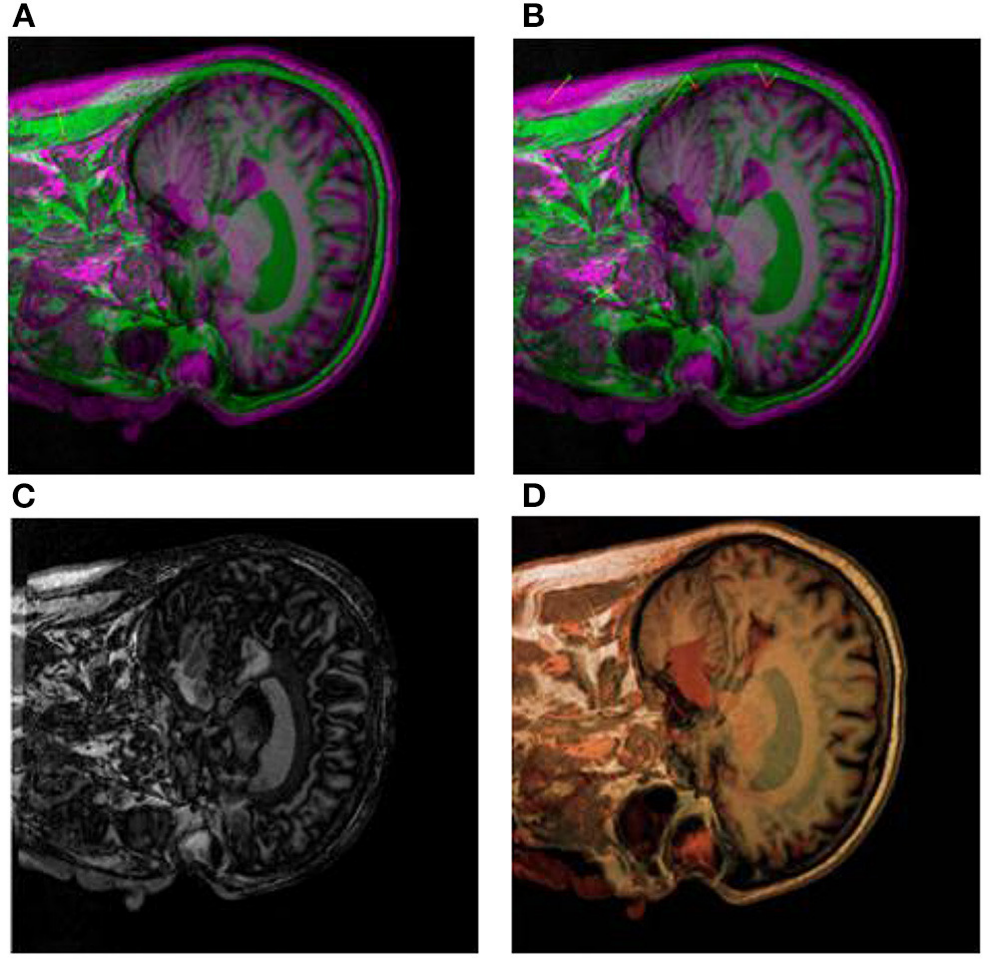
Segmental registration images of brain CT-MRI and PET images. **(A)** shows the pre-processed initial registration image. **(B)** medical images after multi-modal registration. **(C)** is affine transformation global rough registration of medical images. **(D)** represents the medical image after non-rigid transformation and high-precision registration, and the addition of false color is convenient for physicians to identify.

Secondly, we use CT-MRI images as reference images and PET images as floating images for medical registration. In Figures 9A–D are partial registration images of CT-MRI and PET images, respectively. Among them, Figure 9A pre-processed initial registration image. Figure 9B is the medical image after multimodal registration. Figure 9C is a medical image of affine transformation global coarse registration. Figure 9D For the final high-precision registration results, we add false colors to facilitate physician recognition.

## Discussion

In one fusion, that is, when CT images and MRI images are aligned, the mutual information between the two is 0.781. In the second fusion, namely, when the CT-MRI image and PET image are aligned, the mutual information between the two is 0.622, as shown in Table 1.

**Table 1:** Comparison of mutual information fusion methods.

	**First integration of mutual information**	**Second fusion of mutual information**
**PV method**	0.752	0.601
**Proposed NPV method**	0.781	0.622

It can be seen from Table 1 that the proposed NPV method has improved the mutual information of the first and second time compared with the proposed PV method, indicating that this method has higher accuracy in graph registration.

In the process of image diagnosis, it is difficult for any single image to fully display the pathological structure and case characteristics of patients. Different images have prominent advantages for pathological diagnosis, and different imaging equipment, imaging angle and image mode often lead to many differences in diagnosis results. Therefore, reasonable medical image registration is of great significance. In order to verify the new registration algorithm proposed in this paper, CT images, MRI images, and PET images are used as original images for registration. Firstly, image preprocessing (Figures 8A, 9A) and multimodal registration (Figures 8B, 9B) were performed on the above three images. Then, global coarse registration (Figures 8C, 9C), B-spline function solution, image deformation and NPV interpolation, similarity measure analysis and other steps were performed on the first two images to obtain the registration results of CT and MRI images Figure 8D). Thirdly, the registration results were re-registered with PET images, and the secondary operation was completed according to the above steps (Figures 9A–C). The final synthesis results are shown in Figure 9D.

## Conclusion

Medical image registration is an important subject in medical research and is of great significance in medical diagnosis and medical image analysis. This paper presents a new NPV image registration model. The proposed image registration model preprocesses the image before registration, so that the subsequent registration results are more accurate and reliable. The cubic spline interpolation and DFP optimization algorithm are introduced to operate the image. In addition, the proposed method also strictly controls the similarity measure, and ensures that the similarity measure is optimal through multiple iterations. The method has strong operability. By comparing the synthetic image with three original images to be registered, the image registration method proposed in this paper has great advantages in comprehensively displaying pathological details, balancing the advantages of each image, and matching the corresponding spatial points, which plays an important role in clinical application and theoretical research. Through the specific human brain image, compared with the traditional PV image, the sensitivity test shows that the image registration model proposed in this paper has high sensitivity, high reliability and popularization to gray change. Compared with the traditional registration method, this method has higher accuracy, better robustness and is easy to implement. In the direction of face recognition and fingerprint recognition, this model should also have certain reference significance.

However, due to the different imaging principles of medical images in different modes, there may be great differences in attributes. This method is a kind of non-rigid registration, which often requires the design of feature method and the adjustment and optimization of parameters, and it is difficult to obtain a highly universal registration effect. Moreover, although the proposed NPV image registration model is easy to implement, it needs a large amount of calculation and high-performance computer software, which is of little help to the reality of remote areas.

Therefore, our subsequent work is mainly to further improve the algorithm and extend it to other image recognition fields, so that it has higher applicability in areas with normal health conditions.

## Data Availability Statement

The original contributions presented in the study are included in the article/supplementary material, further inquiries can be directed to the corresponding author/s.

## Author Contributions

The author confirms being the sole contributor of this work and has approved it for publication.

## Conflict of Interest

The author declares that the research was conducted in the absence of any commercial or financial relationships that could be construed as a potential conflict of interest.

## Publisher's Note

All claims expressed in this article are solely those of the authors and do not necessarily represent those of their affiliated organizations, or those of the publisher, the editors and the reviewers. Any product that may be evaluated in this article, or claim that may be made by its manufacturer, is not guaranteed or endorsed by the publisher.
